# The Countess of Dufferin's Fund

**Published:** 1889-05-11

**Authors:** Harry Cooper

**Affiliations:** Major


### The Countess of Dufferin's Fund.

### THE NATIONAL ASSOCIATION FOR SUPPLYING FEMALE MEDICAL AID TO THE WOMEN OF INDIA.*

When the Countess of Dufferin, on the eve of her departure for India, took leave of the Queen-Empress in 1884, Her Majesty expressed a wish that something should be done to ameliorate the lot of the women in her Indian empire. On arrival in India, Lady Dufferin found that medical relief was warmly appreciated by the Indian women. Marked success had attended the work of zenana medical missionaries and ladies practising independently, but qualified practitioners and sustained effort were required to give the movement extension and stability.

The idea of a society for promoting female medical relief was heartily welcomed, and in August, 1885, the Queen-Empress having graciously signified her intention of becoming patron, the prospectus of the National Association for Supplying Female Medical Aid to the Women of India was issued.

The new association was to be national and unsectarian, and to consist of life committees (donors of £500), life members (donors of £50), and ordinary members.

The Viceroy became patron in India, and the Countess of Dufferin the first lady president. The organisation was rapidly proceeded with, and by the end of 1886 the association had become fairly established, and branches formed in Bengal, Burar, Burmah, Bombay, the Central Provinces, Madras, Mysore, the North-Western Provinces, and Oudh, and the Punjaub. These branches were virtually independent, and had entire management of their own affairs as long as the general rules of the association were observed. The government was placed in the hands of a central committee, nominated by the lady president, and of the executive committees in the various branches, assisted by local and sub-committees.

In the spring of 1888 the National Association was registered under the Indian Charitable Society Act, XXI., of 1860. All moneys raised in support of it are credited to "the Countess of Dufferin's Fund," and the amount at disposal of the central committee and the branches during 1888 was over ten lakhs of rupees.

When the central committee began work it found that female students were already working in the medical classes at Bombay, Calcutta, Lahore, and Madras; whilst at Agra Dr. Hilson has just succeeded in attaching a female class to the Hospital Assistant School. Medical tuition was the first object recorded on the prospectus of the National Association, and the committee thought that in no way could they further the work better than giving an undivided support to existing schools. Acting on these lines, every effort was made to increase the efficiency of the female medical staff employed at the schools named; to improve the status of Indian female medical students by giving them proper accommodation in lecture-rooms and boarding-houses, and by offering scholarships and prizes. The result is already promising, and at the present moment there are some 200 women studying medicine in the various Indian schools. The majority of these students will never go higher than the Hospital Assistant class, but some are taking university degrees in India, whilst others are going home to perfect their professional education in the schools of England and the Continent. At first, owing to the customs of the country, it was difficult to induce girls of suitable position to come forward as medical students, but that difficulty was overcome. The heads of schools are now in a position to select, and the central committee has pressed upon them the importance of training only such girls as are morally and physically fitted for the high calling they propose following. The success of the vernacular class at Agra induced the Bengal Government to open a similar class for female hospital assistants at the Campbell Hospital, Calcutta; and at Hyderabad (Deccan), the Nizam opened a class for females at the Medical College, where students are given a preparatory medical education, enabling them to finish in Europe or at the Indian medical colleges.

Of the eleven ladies now employed by the National Association, five were educated in Europe or America and six in India. These ladies' professional acquirements vary very much; and it was found that the term lady doctor was supposed to apply equally to all female practitioners, from the certificated nurse to the lady who had gained high honours at a university. To avoid this, it was determined that they should be graded in a manner similar to that applied to men. The title "lady doctor" will in future be only given to those ladies who are entitled to register under the Medical Acts of the United Kingdom—The titles of "female assistant surgeon," and "female hospital assistant" being given to women with corresponding degrees to those held by men in the Government service.

Till funds admitted, the work of the National Association was mostly limited to opening dispensaries and female wards in existing hospitals. But latterly some twelve female hospitals have either been opened or affiliated to the society; indeed, this particular form of female medical relief has gone on as quickly as the number of women qualified to take charge would permit. The head of a large hospital should be a man; but there is no reason why the doctor in charge of the female wards should not be a lady, and all the staff under her should be women. The association aimed at this, and in opening new wards was very anxious that the staff in charge should be one capable of receiving and tending native patients who could not enter the hospital were men about. The women of India who can receive medical treatment from the hands of men are still in a vast minority, and it is for the benefit of the majority that the association was started.

The ignorance of the old *dhais* was such and the malpractices so general that the provision of duly qualified midwives was a benefit immediately appreciated. There was a tendency at first for the trained women to ask exorbitant fees, but this is righting itself, and as the qualified women become more numerous the fees will fall within the means of those poorer families whose pride will not allow them at present to enter a hospital, and whose caste has hitherto prevented them obtaining qualified medical relief owing to absence of trained women. Though most anxious to train as many nurses as quickly as possible, the central committee was careful to endeavour to limit the numbers under tuition at any one place to the number of cases available for practical instruction, and impressed most earnestly on all the importance of the women receiving practical bedside instruction prior to being granted certificates. It was urged that Government should prohibit unlicensed women to practise midwifery, but till the number of certified women is vastly increased, it would be dangerous to attempt legislation on this point; and should it be attempted, for many years to come such legislation could only be applied within limited areas.

No summary of the work of the National Association during its first three years would be complete without an acknowledgment of four very important steps taken by the Government of India in furtherance of the objects of the society:

(i) The introduction into primary schools for girls of "The Way to Health," a sanitary primer used in Southern India, but which, by the courtesy of the Madras publishers, was adapted to the requirements of the Educational Department in other provinces.

* "Record of Three Years' Work." By the Countess of Dufferin. Price 1s. The Annual Report of the National Association for Supplying Female Medical Aid to the Women of India. Price 1s. Hatchards, 97, Piccadilly.

(ii) By drawing the attention of municipalities to their duty in providing female medical aid either in dispensaries, hospitals, or in offering scholarships for female students.
(iii) By allowing the Surgeon-General to help in the selection of candidates in India for employment under the National Association, and for supervising the work of the association in dispensaries, hospitals, and schools.
(iv) In obtaining the sanction of the Secretary of State for candidates at home being examined by an officer of the India Office Medical Board prior to their engagement.

This recognition of the association, though not binding Government to any responsibility, has given position to the employés, secures for the association due execution of its work, and eliminates as far as possible the chance of friction, so liable to occur where highly-trained officials are the servants of voluntary and local committees.

Too much praise cannot be given to the ladies who were practising medicine in India before the association began work. Most of them were missionaries, and to such the objection that the new association was unsectarian might have been serious; but one and all have come forward readily to help with advice and assistance, and it is hardly too much to say that the fortunate avoidance of many difficulties was greatly owing to their kind advice and unselfish co-operation.

The ruling chiefs almost without exception liberally supported the movement, and in many of the feudatory states hospitals for women and children have been built or are now building. These hospitals and dispensaries are one and all maintained by the Durbars, and provided the official in charge performs her duty with judgment and skill their position need give no anxiety. But it is in the selection of the female staff that the National Association at present find some of their chief difficulties. The Durbars, as a rule, ask either for an English lady or an Indian lady doctor. The salary offered is not sufficient to tempt English ladies, and the Indian lady doctors are not yet forthcoming. When women are trained in India to stand professionally alone the difficulty will cease, as Indians (both natives and others) born in the country can accept salaries which it would be impossible for the highly-trained lady doctor from Europe to take.

Wherever female hospitals are attached to medical schools the National Association wishes to see the head of the female staff a lady of high moral and professional acquirements. For such a lady they are prepared to give a salary much higher than those awarded in stations where the work is purely tending the sick or training nurses. Separate female hospitals now exist at Ahmedabad, Bombay, Calcutta, Durbungha, Hyderabad (Deccan), Lahore, Madras, Mysore, Nagpore, C. P., Udaipur, and Ulwar; others are building at Agra, Kapurthala, and Patiala; whilst separate wards for women are opened at Bhopal, Indore, Hyderabad (Sind), Lucknow, and Rangoon, and are proposed at Dacca, Delhi, and many other places. The above are all more or less in connection with the National Association, and they are all working for the same object. The association has by drawing attention to the subject already succeeded in inducing municipalities to come forward liberally, and it is probable that as the machinery of the National Association becomes extended every large city will have its hospital under a lady doctor, and the smaller towns female wards and dispensaries, with assistant surgeons and hospital assistants in charge working under the supervision of the civil surgeons.

But to carry out these plans further funds are necessary, and with a view of bringing the aims of the association more prominently before the public at home, Lady Dufferin is now engaged in organising a corresponding committee in London, who will act as the agents of the central committee in India.

The above account of female medical work in India is very meagre, but full details can be found in the excellent pamphlet written by the first lady president on her departure from India last winter, and in the annual reports of the central committee, published every January at Calcutta.

In conclusion, it only remains to say how much the success of the National Association has been due to the personal exertions of the Marchioness of Dufferin and Ava. Her Excellency first started the scheme, and then organised it. During her residence in India no meeting of the association was held without her presence, and no important document was received or despatched without Lady Dufferin's cognisance. No detail, however trivial, was too small for her attention, and every employé of the association felt that influence which can only be exercised by an intimate acquaintance with each individual case.

Sir Archibald Colvin, the Lieut.-Governor of the N.W. Provinces, in his address to Lord Dufferin on the laying of the foundation-stone of the lying-in ward at Agra last November (1888), said: "I think your excellency must be astonished, as you pass from place to place, to see how thoroughly the idea which emanated first from your mind has communicated itself to others about you, and with what rapidity the little spark which you first lit is spreading itself over the length and breadth of India. I myself should never have thought it possible that in so short a time we should have attracted so many sufficiently educated Hindu and Mohammedan women to our medical classes. That we have done so is proof in itself that the country was ready for the movement. Brilliant as are the achievements of those who have from time to time directed the political doctrines of this country, the great social reform which your excellency has been the first to introduce, will yield to none in the future annals of India."

Harry Cooper, Major.

